# Prevalence of multi-drug resistant (MDR) and extensively drug-resistant (XDR) phenotypes of *Pseudomonas aeruginosa* and *Acinetobacter baumannii* isolated in clinical samples from Northeast of Iran

**DOI:** 10.1186/s13104-020-05224-w

**Published:** 2020-08-10

**Authors:** Bahman Mirzaei, Zahra Norouzi Bazgir, Hamid Reza Goli, Fatemeh Iranpour, Fatemeh Mohammadi, Ryhaneh Babaei

**Affiliations:** 1 Department of Medical Microbiology and Virology, Faculty of Medicine, Mazandaran University of Medical Science, Sari, Iran; 2 Student Research Committee, Faculty of Medicine, Mazandaran University of Medical Sciences, Sari, Iran; 3 Department of Medical Microbiology and Virology, School of Medicine, Zanjan University of Medical Science, Zanjan, Iran

**Keywords:** Multi-drug resistant (MDR), Extensively-drug resistant (XDR), *Pseudomonas aeruginosa*, *Acinetobacter baumannii*, Carbapenem resistant *A. baumannii*, Nosocomial infections

## Abstract

**Objective:**

Multi and extensively drug-resistant (MDR and XDR)*, Pseudomonas aeruginosa* (*P. aeruginosa*) and *Acinetobacter baumannii* (*A*. *baumannii*) are two main causative agents of nosocomial infections leading to increased morbidity and mortality. We aim to study the prevalence of MDR and XDR-*A. baumannii* and *P. aeruginosa* phenotypes in clinical specimens. We conducted this for 1 year (2017–2018) and isolated bacteria from the clinical samples. Then, XDR and MDR strains were determined by susceptibility testing (disc diffusion).

**Results:**

Out of 3248 clinical samples, *A. baumannii* and *P. aeruginosa* strains were detected in 309(9.51%) of them. Susceptibility testing indicated that (16.50%) and (15.53%) of the *P. aeruginosa* and (74.75%) and (73.13%) of the *A. baumannii* isolates were screened as the MDR and XDR strains. The frequency of MDR isolates was higher in wound samples 222 (71.8%). This rate in behavioral intensive care unit (BICU) and restoration ward, were 187 (60.5%) and 63 (20.4%). The frequency of XDR isolates in BICU 187 (59.54%), restoration 58(18.77%), and burns 30 (9.70%) were assessed as well. Considering high isolation rates of MDR and XDR of mentioned strains, it is necessary to apply prevention criteria for eradication of the mentioned bacteria from hospital wards.

## Introduction

In recent years, hospital-acquired infections have become one of the most common causes of mortality and morbidity [1–3]. Due to the increasing rates of antibiotic resistant Gram-negative infections, selection of therapeutic options against mentioned infections is restricted [4]. In Europe, published studies estimates that resistant bacterial healthcare-acquired infections account for an estimated nearly 400,000 patients each year. [5–7]. Mortality rates of *P. aeruginosa* as a frequent causative agent of 10–15% of nosocomial infections have been estimated to range from 18 to 61% [8, 9]. Recently, increasing of the MDR—*A. baumannii* as another non-fermentative Gram-negative bacilli in nosocomial infections has been demonestrared [8]. XDR bacterium associated infections treatment, because of limitations to effective antimicrobial agents administration, is still challenging and lead to significant infection control problems [10, 11]. Rates of MDR—*P. aeruginosa* are increasing in United States of America (4% in 1993 to 14% in 2002 and Italy (2.1% in 2007 to 4.1% in 2010) [4]. Moreover, MDR *P*. *aeruginosa* account for 13–19% of hospital acquired infections (HAIs) each year in the US. Mortality rates between 2007 and 2009 was estimated 46.1%) [12, 13]. MDR—*P. aeruginosa* isolates in South America and Malaysia were estimated as 8.2% and 6.9%, as well [4]. Throughout last decades, MDR—*A. baumannii* phenotypes due to antibiotic resistant characteristics, environmental durability, and dissemination capability within health care facilities are assigned as morbidity and mortality related infection causative agent [14]. As example, mortality rate of MDR—*A. baumannii* in bacteremia was estimated to be 21.2% [15]. Mortality ranging from 5% in general wards to 54% in intensive care units (ICUs) is associated with *A. baumannii* infections [11, 16]. Cleaning and observing disinfection procedures are the main approaches to prevent nosocomial infections, otherwise, the rate of antibiotic—resistant bacteria would be constantly increased making the treatment process problematic, which may lead to longer duration of hospitalization, increased treatment costs, and also higher rates of mortality [17–19]. Accordingly, regarding the undeniable role of MDR and XDR—*P. aeruginosa* and *A. baumannii* isolates in the development of hospital acquired infections and their high resistance to several antibiotics, the present study was conducted to evaluate the prevalence of mentioned phenotypes and their antibiotic susceptibility patterns.

## Main text

### Materials and methods

#### Samples collection and culture

In the current cross-sectional study, a total of 3248 clinical samples including wound, urine, sputum, blood, feces, and trachea were collected from January 2017 to December 2018 from a burn center hospital, northeast of Iran. Samples were cultured on selective media including MacConkey agar, and Thioglycollate broth, by Liofilchem, Italy (24 h on 37 °C) based on bacteriology standards.

#### Identification of Bacterial Isolates

Isolates were identified based on the Bergeys̓ microbiology book guidelines [20]. In brief, conventional biochemical tests including oxidase, catalase, motility, metabolic procedure such as citrate, Indol production, Methyl red, Voges–Proskauer, and presence of lysine decarboxylase, and arginine dehydrogenase enzymes were performed.

#### Re-confirmation of *P. aeruginosa* and *A. baumannii* isolates by PCR

Molecular identification of *P. aeruginosa* and *A. baumannii* was performed by targeting the gene for *P. aeruginosa* and *A. baumannii* isolates using specifically designed primers (*P. aeruginosa 16sDNA* and *blaOXA*-*51* to *A. baumannii*) which produced a 956 and 353 bp PCR product [21, 22]. Amplification was done based on the mentioned references [21, 22] with slight modification on the Gene Amp PCR system (Applied Biosystem, USA) in the total volume of 25 μl containing 14 μl master amplicon (Biolab, New England, UK), 1 pmol of each forward and reverse primers, a minor amount of colony as a template and 9 μl distilled water. The first cycle of denaturation was at 95 °C for 5 min, followed by 25 cycles at 94 °C for 1 min, then at 58 °C, (55 for *blaoxa51*) for 1 min, at 72 °C for 60 s, and finally a terminal extension for 5 min. PCR products were visualized by 1% agarose gel (KBC, Max Pure agarose, Spain) containing 0.5 µg/mL DNA Safe Stain dye in gel image analysis system (UVitec, Cambridge, UK). A*. baumannii* ATCC 19606 and *P. aeruginosa* ATCC 27853 were used as the positive and negative control strains, respectively.

#### Antibiotic susceptibility tests

Isolates confirmed as *P. aeruginosa* and *A. baumannii* by biochemical and molecular tests underwent the disc diffusion susceptibility test based on the clinical and laboratory standards institute (CLSI, M100S 26th edition breakpoints) guidelines [23]. In short, a 0.5 McFarland suspension of each isolate was inoculated on a whole plate surface Mueller–Hinton agar plate by streaking the swab in back and forth motions. Antimicrobial impregnated discs including amikacin (30 μg), ceftazidime (30 μg), cephalexin (30 μg), ciprofloxacin (5 μg), imipenem (10 μg), meropenem (10 μg), gentamycin (10 μg), tobramycin (10 μg), and cotrimoxazole (25 μg) were put on the surface of the agar, and the plates were incubated for 24 h at 37 °C. Following incubation, inhibition zone sizes to the nearest millimeter were measured using a ruler. Using published CLSI guidelines, susceptibility, or resistance of the organism to each tested drug was determined. Interpretation of Antibiotic susceptibility to evaluated MDR and XDR isolates was performed based on the european centre for disease prevention and control (ECDC) instructor as well [24].

#### Statistical analysis

In this study, we are using a Chi squared test, (χ^2^ test). Statistical analysis of results was accomplished by using SPSS version 19 and a *P* value < 0.05 was considered significant.

### Results

309 (9.51%) *P. aeruginosa* and *A. baumannii* isolates from clinical-samples were collected in a main center (Zareh Burn Hospital). 234 (75.7%) and 75 (24.3%) were identified respectively as *A. baumannii* and *P. aeruginosa*, using biochemical tests. All the isolates were then reconfirmed by PCR.

Isolates were collected from patients with mean age of 49.8 years old. Out of 309 obtained isolates, 246 (79.6%) of them were from wound, 29 (9.4%) of them were from urine, and 24 (7.8%) and 10 (3.2%) of them were from blood and sputum, respectively. Susceptibility testing indicated that, 48 (15.53%) and 23 (7.44%) of the *P. aeruginosa* isolates were screened as imipenem resistant and susceptible strains. The mentioned rates of the isolated *A. baumannii* against imipenem were 228 (97.4%) and 5 (2.1%). 228(97.4%) of *A. baumannii* isolates and 47 (62.7%) of *P. aeruginosa* isolates were resistant against ciprofloxacin, the data showed. Detailed data are listed in Table 1.

**Table 1:** Antibiotic susceptibility patterns of isolated *P. aeruginosa* and *A. baumannii* strains

Antibiotic susceptibility pattern	*P. aeruginosa n*(%)			*A. baumannii n(%)*		
	S	R	I	S	R	I
Amikacin	36 (48%)	39 (52%)	0	14 (6%)	208 (88.9%)	12 (5.1%)
Gentamycin	35 (46.7%)	39 (52%)	1 (1.3%)	–	234 (100%)	–
Tobramycin	23 (30.7)	47 (62.7%)	5 (6.7%)	5 (2.1%)	227 (97%)	2 (0.9%)
Imipenem	23 (30.7)	48 (64%)	4 (5.3%)	5 (2.1%)	228 (97.4%)	1 (0.4%)
Meropenem	24 (32)	45 (60%)	6 (8%)	5 (2.1%)	226 (96.6%)	3 (1.3%)
Ceftazidime	30 (40)	44 (58.7%)	1 (1.3%)	8 (3.4%)	226 (96.6%)	–
Cephalexin	5 (6.7)	70 (93.3%)	–	8 (3.4%)	296 (96.6%)	–
Ciprofloxacin	27 (36)	47 (62.7%)	1 (1.3%)	6 (2.6%)	228 (97. 4%)	–
Cotrimoxazole	16 (21.3)	59 (78.7%)	–	4 (1.7%)	224 (95.7%)	6 (2.6%)

*N* number of isolated strains,  % percentage of susceptibility patterns, *S* sensitive, *R* resistant, *I* intermediate

According to the susceptibility testing results, 231 (74.75%) of *A. baumannii* and 51(16.50%) *P. aeruginosa* isolated strains were categorized as MDR strains. The prevalence of XDR—*A. baumannii* and *P. aeruginosa* strains were 226 (73.13%) and 48 (15.53%), respectively (Table 2).

**Table 2:** Prevalence of MDR and XDR isolated strain according to the gender

Strains	MDR strains		XDR strains	
*P. aeruginosa*	51 (16. 50%)		48 (15.53%)	
	M: 37 (72.5%)	F: 14(27.5%)	M: 35 (72.91%)	F: 13 (27%)
*A. baumannii*	231 (74.75%)		226 (73.13%)	
	M: 155 (67.1%)	F: 76(32.9%)	M: 152 (67.3%)	F: 74 (32.7%)

The percentage of isolated strains was assessed in 309 total number of *P. aeruginosa* and *A. baumannii* isolates

*MDR* multi-drug resistant, *XDR* extremely- drug resistant, *M* male, *F* female

The frequency of MDR isolates in this main center was higher in wound samples 222 (71.8%).MDR strains in behavioral intensive care unit (BICU) 187 (60.5%) and restoration ward 63 (20.4%) more isolated than other hospital wards. The frequency of XDR isolates in BICU 184 (59.54%), restoration 58 (18.77%), and burns 30 (9.70%) were assessed as well. Detailed data according to the age/ward and clinical specimens of MDR and XDR isolates are listed in Additional files 1 and 2.

Considering the relationship between age and sex with resistance to different antibiotics, only aminoglycoside and meropenem resistant strains were statistically significant (*P *< 0.05).

### Discussion

Given the overuse of antibiotics in hospitals and constant rise in antibiotic resistance, to prevent new resistant strains appearance, evaluation of resistant isolates by susceptibility testing seems to be critical. Due to genetic alteration caused by unnecessarily prescribed antibiotics, resistance patterns could be different in each countries region [25]. Over the recent years, various articles have confirmed an increasing MDR features among *P. aeruginosa* isolates from burn hospitals [26]. Based on a previously published study [27] on *P. aeruginosa* in a burn center, high resistance patterns against ciprofloxacin (93.7%), and amikacin (82%) were observed. In the 79.2% of *P. aeruginosa* isolates, imipenem resistant pattern was observed. In a study conducted in 2013 at a burn hospital center of Gilan province in the northwest of Iran, the percentage of resistance to tested antibiotics was as follows; ceftazidime 57.5%, ciprofloxacin 65%, gentamycin 67.5%, piperacillin 87.5%, amikacin 90%, and imipenem 97.5%. In the current study, the frequency of the ciprofloxacin and amikacin resistant *P. aeruginosa* isolates were (62.7%) and (52%), respectively. Sixty-four percent of *P. aeruginosa* isolates was imipenem resistant, as well [27]. Corehtash reported that 93.1% of isolated strains were MDR-*P. aeruginosa* [27].

Based on the Nasimmoghadas et al., findings, *P. aeruginosa* isolates were almost resistant to all tested antibiotics, except polymixin B (2%) and ceftazidime (32%). Ninety-four percent and 85% of isolates of the mentioned study were assigned as MDR and XDR strains [24]. At the current research, MDR and XDR-*P. aeruginosa* isolates were estimated as 16.50% and 15.53% (n = 309). Other researchers found that totally, 45.3%, 30.1% and 5.46% of the isolates were MDR as well [28].

Preze et al., in 2019 by collecting fifty-nine *P. aeruginosa* from twelve different hospitals in Spain, Italy and Greece indicated that the prevalence of the XDR and MDR isolates in Greece samples was 88.9%. Totally, 19 (35.8%) *P. aeruginosa* isolates were XDR phenotype and 16 (30.2%) isolates were screened as MDR strains [29]. Another published study conducted by Saleem et al., in Pakistan which reported that, out of 88 *P. aeruginosa* isolates, 30.2%, 17.4% and 37.2% of them were resistant to imipenem, ciprofloxacin, and amikacin. Also, prevalence of MDR (36.3%) and XDR (18.1%) isolated strains were assessed [30]. The range of imipenem and ciprofloxacin resistant patterns in the current research were almost twice as the Saleem findings. The frequency of MDR and XDR isolates in our results were higher than the mentioned study too. There is a difference between our results and mentioned study findings. Improper antibiotic prescriptions in our hospital could be a possible reason for this variation.

Resistance to antibiotics in *A. baumannii* has reached alarming levels worldwide, particularly for carbapenems [10]. At the current study, tobramycin, ceftazidime, ciprofloxacin, and imipenem resistant *A. baumannii* were screened as follow; (97%), (96.6%), (97.4%) and (97%). MDR-*A. baumannii* and XDR-*A. baumannii* frequencies, in a published study in 2018, was 84% and 48% [31]. According to Hatami’s findings, up to 70% of *A. baumannii* isolates were resistant to tobramycin and ceftazidime. In addition, up to 50% and 80% of isolates were resistant to amikacin and imipenem [32]. Our findings to MDR and XDR-*A. baumannii* isolates revealed that, up to 89% of isolates were MDR (89.32%) and XDR (91.90%). MDR and XDR-*A. baumannii* frequencies in another published study were 83.9% and 16.1% [32]. Probably due to conducting improper infection control strategies in hospitals, the rate of resistance of *A. baumannii* against imipenem and meropenem has increased and become a major concern worldwide. Based on a previously published [32] study, all *A. baumannii* isolates were resistant to imipenem. This amount to meropenem was reported as 99.2% as well. According to the conducted study in Brazil in 2014, resistance rates of carbapenem resistant *A. baumannii* has been evaluated to be high (80.7%) [33]. Rossi and her colleagues by performing a cross-sectional study in Brazil in 2017 reported that between 2010 and 2014 a variation of 30% to 70% in carbapenem resistant *A. baumannii* was observed [34]. Romanin in 2019, by performing a study on 103 MDR-*A. baumannii* showed that carbapenem resistant *A. baumannii* isolates were the main part of MDR isolated strains (92.2%). The prevalence of XDR-*A. baumannii* strains in the mentioned study was estimated at 78.6% as well [35]. Our *A. baumannii* isolates demonstrated a high resistant pattern against carbapenems antibiotics as well (97%). The results presented similarity with the latest screenings of Brazilian researchers. Our finding confirms the observed data of the mentioned studies. In conclusion, high prevalence of MDR and XDR—*P. aeruginosa* and *A. baumannii* strains in the northeast of Iran regions is a serious concern in hospital wards. These findings highlight the need for hasty identification implement strict antimicrobial stewardship policies and strong microbiological surveillance procedures in the hospitals.

## Limitation

Responsible genes to antibiotic resistance and genetic relationship between the resistant strains are not determined and these are the limitation of this study.

## Supplementary information

**Additional file 1.** Frequency of MDR—*P. aeruginosa* and *A. baumannii* isolates regarding to the age, wards and clinical samples.

**Additional file 2.** Frequency of XDR—*P. aeruginosa* and *A. baumannii* isolates regarding to the age, wards and clinical samples.

## Data Availability

All the results of this study have been classified and maintained by the dissertation in the Mazandaran University of medical science. We have indeed provided all raw data on which our study is based. Competing Interests: The authors declare that they have no competing interests. All data generated or analysed during this study are included in this published article [and its supplementary information files.

## Abbreviations

MDR and XDR: Multi and extensively drug-resistant
*P. aeruginosa*: *Pseudomonas aeruginosa*
*A*. *baumannii*: *Acinetobacter baumannii*
BICU: Behavioral intensive care unit
HAIs: Hospital acquired infections
ICUs: Intensive care units
CLSI: Clinical and laboratory standards institute
ECDC: European Centre for Disease Prevention and Control
