# Supplementary material for: Prevalence of multi-drug resistant (MDR) and extensively drug-resistant (XDR) phenotypes of Pseudomonas aeruginosa and Acinetobacter baumannii isolated in clinical samples from Northeast of Iran
Source: BMC Res Notes. 2020 Aug 10;13:380. doi: 10.1186/s13104-020-05224-w (PMC7418330; doi:10.1186/s13104-020-05224-w)
Supplement: Supplementary file 1 — Additional file 1. Frequency of MDR—P. aeruginosa and A. baumannii isolates regarding to the age, wards and clinical samples. [file 13104_2020_5224_MOESM1_ESM.docx]

**Additional file 1**: Frequency of MDR *P. aeroginosa* and *A. baumannii* isolates regarding to the age, wards and clinical samples.

| **Wards and clinical specimens**  **n (%)** | | **BICU** | **Out patients** | **Burn** | **Restoration** | **Psychology** | **Wound** | **Urine** | **Sputum** | **Blood** |
| --- | --- | --- | --- | --- | --- | --- | --- | --- | --- | --- |
| **Age** | **Is** |  |  |  |  |  |  |  |  |  |
| 0-1 | PA  AB | 2 (66.7)  23 (82.1) | -  - | 1 (33.3)  5 (17.9) | -  - | -  - | 2 (66.7)  23(82.1) | 1 (33.3)  1 (3.6) | -  1 (3.6) | -  3 (10.7) |
| *11-20* | PA  AB | 3 (60)  12 (85.7) | -  - | -  - | 2 (40)  2 (14.3) | -  - | 2 (40)  11(78.6) | 1 (20)  3 (21.4) | -  - | 2 (40)  - |
| 21-30 | PA  AB | 2 (66.7)  33 (76.7) | -  - | -  7 (16.3) | 1 (33.3)  3 (7) | -  - | 1 (33.3)  32(74.4) | 1 (33.3)  8 (18.6) | -  2 (4.7) | 1 (33.3)  1 (2.3) |
| 31-40 | PA  AB | 6 (85.7)  19 (50) | -  - | -  4 (10.5) | 1 (14.3)  15(39.5) | -  - | 6 (85. 7)  35 (92.1) | -  1 (2.6) | -  2 (5.3) | 1 (14.3)  - |
| 41-50 | PA  AB | 8 (61.5)  20 (69) | -  - | 2 (15.4)  4 (13.8) | 2 (15.4)  5 (17.2) | 1 (7.7) | 8 (61.5)  23(79.3) | 2 (15.4)  4 (13.8) | 1 (7.7)  - | 2 (15.4)  2 (6.9) |
| 51-60 | PA  AB | 4 (36.4)  26 (68.4) | --  - | 1 (9.1)  1 (2.6) | 6 (54.5)  11(28.9) | -  - | 11 (100)  28(73.7) | -  3 (7.9) | -  2 (5.3) | -  5 (13.2) |
| 61-70 | PA  AB | -  9 (56.2) | -  1 (6.2) | 2 (25)  1 (6.2) | 6 (75)  5 (31.2) | -  - | 7 (87.5)  13(81.2) | 1 (12.5)  - | -  - | -  3 (18.8) |
| 71-80 | PA  AB | 1 (100)  10 (71.4) | - | -  - | -  4 (28.6) | -  - | -  11(78.6) | -  - | 1(100)  - | -  3(21.4) |
| 81-90 | PA  AB | -  9 (81.8) | -  - | -  2 (18.2) | -  - | -  - | -  9 (81.6) | -  1 (9.1) | -  - | -  1 (9.1) |
| **Total N (%)** |  | 187 (66.3) | 1 (0.4) | 30(10.6) | 63(22.3) | 1 (0.4) | 222 (78.7) | 27 (9.6) | 9 (3.2) | 24 (8.5) |

PA; *P. aeruginosa*, AB; *A. baumannii*, Is; isolated strains.
