# Supplementary material for: Prevalence of multi-drug resistant (MDR) and extensively drug-resistant (XDR) phenotypes of Pseudomonas aeruginosa and Acinetobacter baumannii isolated in clinical samples from Northeast of Iran
Source: BMC Res Notes. 2020 Aug 10;13:380. doi: 10.1186/s13104-020-05224-w (PMC7418330; doi:10.1186/s13104-020-05224-w)
Supplement: Supplementary file 2 — Additional file 2. Frequency of XDR—P. aeruginosa and A. baumannii isolates regarding to the age, wards and clinical samples. [file 13104_2020_5224_MOESM2_ESM.docx]

**Additional file 2:** Frequency of XDR PA and AB isolates regarding to the age, wards and clinical samples.

| ***Wards and clinical samples***  ***n (%)*** | | **BICU** | **Out patients** | **Burn** | **Restoration** | **Psychology** | **Wound** | **Urine** | **Sputum** | **Blood** |
| --- | --- | --- | --- | --- | --- | --- | --- | --- | --- | --- |
| ***Age*** | ***Is*** |  |  |  |  |  |  |  |  |  |
| **0-10** | PA  AB | 2 (66.7)  22(81.5) | -  - | 1 (33.3)  5 (18.5) | - | -  - | 2 (66.7)  23(85.2) | 1 (33.3)  1 (3.7) | -  1 (3.7) | -  2 (7.4) |
| **11-20** | PA  AB | 3 (75)  12 (85.7) | -  - | -  - | 1 (25)  2 (14.3) | -  - | 1 (25)  11(78.6) | 1 (25)  3 (21.4) | -  - | 2 (50)  - |
| **21-30** | PA  AB | 2 (66.7)  32 (76.2) | -  - | -  7 (16.7) | 1 (33.3)  3 (7.1) | -  - | 1 (33.3)  32(76.2) | 1 (33.3)  8 (19) | -  1 (2.4) | 1 (33.3)  1 (2.4) |
| **31-40** | PA  AB | 6 (85.7)  19 (50) | -  - | -  4 (10.5) | 1 (14.3)  15(39.5) | -  - | 6 (85. 7)  35(92.1) | -  1 (2.6) | -  2 (5.3) | 1 (14.3)  - |
| **41-50** | PA  AB | 9 (64.3)  20 (69) | -  - | 2 (14.3)  4 (13.8) | 2 (14.3)  5 (17.2) | 1 (7.1) | 8 (57.1)  23(79.3) | 2 (14.3)  4 (13.8) | 2 (14.3)  - | 2 (14.3)  2 (6.9) |
| **51-60** | PA  AB | 4 (40)  26 (70.3) | -  - | 1 (10)  1 (2.7) | 5 (50)  10(27) | -  - | 10 (100)  27 (73) | -  3 (8.1) | -  2 (5.4) | -  5 (13.5) |
| **61-70** | PA  AB | -  9 (56.2) | -  1 (6.2) | 2 (28.6)  1 (6.2) | 5 (71.4)  5 (31.2) | -  - | 7 (100)  13(81.2) | -  - | -  - | -  3 (18.8) |
| **71-80** | PA  AB | 1 (100)  10 (76.9) | -  - | -  - | -  3 (23.1) | -  - | -  10(76.9) | -  - | -  - | -  3 (23.1) |
| **81-90** | PA  AB | -  8(80) | -  - | -  2 (20) | -  - | - | -  8 (80) | -  1 (10) | -  - | -  1 (10) |
| Total N (%) | | 184 (67.2) | 1 (0.4) | 30(10.9) | 58(21.2) | 1 (0.4) | 217 (79.2) | 26 (9.5) | 8 (2.9) | 3 (8.4) |

**PA; *P. aeruginosa*, AB; *A. baumannii*, Is; isolated strains.**
